# Comprehensive Evaluation and Implementation of Improvement Actions in Butcher Shops

**DOI:** 10.1371/journal.pone.0162635

**Published:** 2016-09-12

**Authors:** Gerardo A. Leotta, Victoria Brusa, Lucía Galli, Cristian Adriani, Luciano Linares, Analía Etcheverría, Marcelo Sanz, Adriana Sucari, Pilar Peral García, Marcelo Signorini

**Affiliations:** 1 IGEVET - Instituto de Genética Veterinaria “Ing. Fernando N. Dulout” (UNLP-CONICET LA PLATA), Facultad de Ciencias Veterinarias UNLP, La Plata, Argentina; 2 Laboratorio de Microbiología de Alimentos, Facultad de Ciencias Veterinarias UNLP, La Plata, Argentina; 3 Departamento de Seguridad Alimentaria, Municipalidad de Berisso; 4 Centro de Investigación Veterinaria Tandil (CIVETAN), CONICET, CICPBA, Facultad Ciencias Veterinarias, UNCPBA; 5 Centro Estudios Infectológicos “Dr. Daniel Stamboulian”, División Alimentos, Ciudad Autónoma de Buenos Aires, Argentina; 6 CONICET - EEA Rafaela, Instituto Nacional de Tecnología Agropecuaria (INTA), Santa Fe, Argentina; Agricultural University of Athens, GREECE

## Abstract

Foodborne pathogens can cause acute and chronic diseases and produce a wide range of symptoms. Since the consumption of ground beef is a risk factor for infections with some bacterial pathogens, we performed a comprehensive evaluation of butcher shops, implemented improvement actions for both butcher shops and consumers, and verified the impact of those actions implemented. A comprehensive evaluation was made and risk was quantified on a 1–100 scale as high-risk (1–40), moderate-risk (41–70) or low-risk (71–100). A total of 172 raw ground beef and 672 environmental samples were collected from 86 butcher shops during the evaluation (2010–2011) and verification (2013) stages of the study. Ground beef samples were analyzed for mesophilic aerobic organisms, *Escherichia coli* and coagulase-positive *Staphylococcus aureus* enumeration. *Salmonella* spp., *E*. *coli* O157:H7, non-O157 Shiga toxin-producing *E*. *coli* (STEC), and *Listeria monocytogenes* were detected and isolated from all samples. Risk quantification resulted in 43 (50.0%) high-risk, 34 (39.5%) moderate-risk, and nine (10.5%) low-risk butcher shops. Training sessions for 498 handlers and 4,506 consumers were held. Re-evaluation by risk quantification and microbiological analyses resulted in 19 (22.1%) high-risk, 42 (48.8%) moderate-risk and 25 (29.1%) low-risk butcher shops. The count of indicator microorganisms decreased with respect to the 2010–2011 period. After the implementation of improvement actions, the presence of *L*. *monocytogenes*, *E*. *coli* O157:H7 and *stx* genes in ground beef decreased. *Salmonella* spp. was isolated from 10 (11.6%) ground beef samples, without detecting statistically significant differences between both study periods (evaluation and verification). The percentage of pathogens in environmental samples was reduced in the verification period (*Salmonella* spp., 1.5%; *L*. *monocytogenes*, 10.7%; *E*. *coli* O157:H7, 0.6%; non-O157 STEC, 6.8%). Risk quantification was useful to identify those relevant facts in butcher shops. The reduction of contamination in ground beef and the environment was possible after training handlers based on the problems identified in their own butcher shops. Our results confirm the feasibility of implementing a comprehensive risk management program in butcher shops, and the importance of information campaigns targeting consumers. Further collaborative efforts would be necessary to improve foodstuffs safety at retail level and at home.

## Introduction

Foodborne diseases are caused by ingestion of foodstuffs contaminated with microorganisms or chemicals, and are considered a growing public health problem worldwide [1]. The World Health Organization estimates that 1,800 million diarrhea episodes and 3 million of deaths in children under the age of 5 occur every year in the world, mainly by contaminated foodstuffs. Also, approximately 75% of new human infectious diseases are caused by pathogens of animal origin and animal products [2]. Foodborne pathogens are the cause of acute and chronic diseases [2, 3]. In Argentina, the incidence of hemolytic uremic syndrome (HUS) is high. Shiga toxin-producing *Escherichia coli* (STEC) were identified as the primary etiological agent, *E*. *coli* O157:H7 being the predominant serotype isolated [4]. Ground beef consumption is a risk factor for infection with several foodborne pathogens, including *E*. *coli* O157:H7, non-O157 STEC and *Salmonella* [5–7].

Food contamination with microorganisms may occur at any stage in the process from food production to consumption, and may be the result of environmental contamination. Cross-contamination of food with pathogens in the retail environment is a significant public health issue that contributes to an increased risk of foodborne illness [8]. Some pathogenic bacteria such as *L*. *monocytogenes*, *Salmonella* spp. or *E*. *coli* O157:H7 have the ability to attach onto stainless and other food-contact surface materials [9–11]. Foodstuff manufacture equipment and the surrounding environment may serve as potential reservoirs of contamination [12, 13].

The comprehensive knowledge of the current status of butcher shops, including the bacteriological analysis of food and environmental samples, risk assessment and handler training could improve the microbiological quality of ground beef sold at retail markets. Microbiological analysis at the verification step helps to determine the impact of improvement actions [14]. In addition, educational campaigns targeting food workers and consumers may play an important role in the prevention of foodborne illness [15, 16].

The aims of this work were therefore a) to perform a comprehensive evaluation of butcher shops, including risk quantification and determination of the bacteriological quality in raw ground beef and environmental samples; b) to implement improvement actions for both butcher shops and consumers; and c) to verify the impact of such improvement actions.

## Materials and Methods

In October 2010, a pilot program called “Healthy Butcher Shops” was conducted in the city of Berisso (34°52′00″S 57°52′00″O), Buenos Aires, Argentina. Berisso has 135 km^2^ and 83,123 inhabitants. A total of 110 butcher shops were identified at the beginning of the program and, from this total, 86 butcher shops completed the program and were included in this study. Beef was provided to butcher shops by ten abattoirs in the region, as follows: abattoir “A”, 48 (55.8%) butcher shops; abattoir “B”, 14 (17.4%); abattoir “C”, 7 (8.1%); abattoirs “D”, “E” and “F”, three (3.5%) each; abattoirs “G”, “H” and “I”, two (2.3%) butcher shops each; and abattoir “J”, one shop. Sampling was randomly performed and covered all the geographic areas of the city. Comprehensive evaluation and risk quantification using a checklist were made at each butcher shop. In addition, six samples from each butcher shop were collected for bacteriological analysis, including ground beef, meat tables, knives, meat mincing machines and manipulator hands. Two of the 86 shops, were supermarkets selling commercially packaged ground beef; therefore, environmental samples were not taken. Ground beef samples presented the organoleptic and commercial characteristics established in the Argentine Food Code (AFC) [17]. They were kindly provided by each butcher shop to carry out the "Healthy Butcher Shops” Program, in full agreement with the Berisso sanitary authorities. Results of this evaluation period were delivered to the person in charge of each butcher shop. Thereafter, a training plan was designed using those results as starting point to implement the improvement actions. Consumers received information about foodborne disease prevention. We also delivered workshops for teachers of all kindergartens in the city. Finally, during the verification period (2013), all butcher shops were re-evaluated using the same tool for risk quantification and the same bacteriological analysis to verify the impact of the improvement actions implemented.

The entire study period lasted from 2010 to 2013. Field work was authorized by the health and supervision authorities of Berisso. All butchers were invited to participate voluntarily; 86 butcher shops completed the program and were included in this study. To this end, a cooperation agreement between the Berisso city authorities and the National University of La Plata School of Veterinary Sciences was signed.

### Risk quantification and sample collection

The checklist used for risk quantification included five groups of variables (total value, 100): 1) situation and conditions of the building (10.0), 2) equipment and tools (15.0); 3), handlers (25.0), 4) raw materials and products for sale (20.0), and 5) production flow (30.0) [18]. Risk assessment on a 1–100 scale was quantified as high-risk (1–40), moderate-risk (41–70), or low-risk (71–100).

A total of 172 raw ground beef and 672 environmental samples, including 168 taken from meat tables, 168 from knives, 168 from mincing machines and 168 from manipulator hands, were collected from 86 butcher shops during the study period (evaluation and verification stages).

From October 2010 to July 2011 (evaluation stage), 86 butcheries were visited. In order to obtain a comprehensive evaluation during the visits, 86 ground beef and 336 environmental samples were taken, and risk quantification was performed. All samples were collected during the day (operational process) before the sanitation step. One kilogram of ground beef was collected in a plastic bag provided by the butcher, under the same conditions as those used for selling the product. Environmental samples were obtained from meat contact surfaces using a sterile sponge (Whirl-Pak speci-sponge, Nasco, USA) soaked in 10 ml of buffered peptone water (BPW) (Biokar, Zac de Ther, France), according to the following protocol. In meat tables, three areas of 20 x 20 cm each (a total of 1,200 cm^2^) were sampled. The sponge was wiped 10 times over each sampling area. The entire surface of the knife blade and the intersection between the blade and the blade handle were sponged. The meat mincing machine was disassembled and the sample was taken from the meat container, the worm meat grinder and the screw ring. In the case of manipulator hands, the sterile sponge sampled all hand surfaces, including front, back, interdigital spaces and nails. All samples were ice- refrigerated and sent to the laboratory to be analyzed immediately.

### Bacteriological analysis

Ground beef samples were analyzed for mesophilic aerobic organisms, *Escherichia coli* and coagulase-positive *Staphylococcus aureus* enumeration [19–21]. All media were from Biokar Diagnostics (Beauvais, France). The AFC [17] microbiological criteria for fresh ground beef were used, *i*.*e*.,: mesophilic aerobic organisms (n:5 c:3 m:10^6^ M:10^7^), *E*. *coli* (n:5 c:2 m:100 M:500), and coagulase-positive *S*. *aureus* (n:5 c:2 m:100 M:1000). Moreover, *Salmonella* spp., *E*. *coli* O157:H7, non-O157 STEC and *Listeria monocytogenes* were detected in and isolated from ground beef and environmental samples. All environmental sponges were aseptically divided into four portions and each sponge portion was used to analyze the different bacteria. In addition, 1 ml BPW from each bag containing the manipulator hand sponge was used for *S*. *aureus* isolation.

#### *Salmonella* spp

Twenty-five g of ground beef and one portion of the sponge from each environmental sample were cultured in 225 ml and 100 ml of lactose broth (Biokar Diagnostics), respectively, for 24±2 h at 35°C [22]. After the pre-enrichment step, 0.1 ml of the broth was put onto 10 ml of Rappaport Vassiliadis broth (Biokar Diagnostics), and 1 ml onto 10 ml of Tetrathionate broth (Acumedia Manufacturers, US) for 24±2 h at 42±0.2°C and 43±0.2°C, respectively. Ten microliters were plated into bismuth sulfite agar (Becton Dickinson, Le Pont de Claix, France), xylose lysine desoxycholate plus tergitol agar (Oxoid, Basingstoke, UK), and hektoen enteric agar (Laboratorios Britania, Buenos Aires, Argentina) and incubated for 24±2 h at 35°C. Two or more presumptive colonies from each plate were studied by biochemical tests. The isolates identified as *Salmonella* spp. were characterized by ribotyping using RiboPrinter^®^ System (DuPont, Wilmington, DE, USA).

#### *Escherichia coli* O157:H7

Sixty-five g of ground beef samples and one portion of sponge from each environmental sample were incubated onto 585 ml and 100 ml of modified Trypticase Soy Broth (Acumedia), respectively, for 20 h at 41.5°C [23]. After enrichment, a specific O157 concentration was made using immunomagnetic separation (Dynal Biotech, Oslo, Norway), streaked into SD-39 agar (Acumedia) and cefixime-tellurite MacConkey sorbitol agar (Oxoid, Hampshire, UK), and incubated for 20 h at 37°C. After incubation, presumptive colonies were selected and screened for *rfb*_O157_, *stx*_1_ and *stx*_2_ genes by multiplex-PCR [24]. The characterization was made by biochemical tests [23] and genotypic profile: *fli*C_h7_, *stx*_1_, *stx*_2_, *ehxA*, and *eae* [25].

#### Non-O157 STEC

Twenty-five g of ground beef samples and one portion of sponge from each environmental sample were incubated in 225 ml and 100 ml, respectively, of modified *Escherichia coli* broth (Acumedia) for 20 h at 41.5°C. SYBR-PCR screening [26] was used after the enrichment step. One milliliter from all RT-PCR-positive samples was plated onto MacConkey agar (Becton Dickinson Co., Sparks, MD, USA) and Levine-Eosyne Methylene Blue agar (Biokar). All plates were incubated for 18 h at 37°C. Fifty colonies with *E*. *coli* morphology were selected from each plate and point-inoculated on nutrient agar (Laboratorios Britania). After incubation, five pools of 10 colonies were screened for *stx*_1_ and *stx*_2_ genes by multiplex-PCR [24]. Colonies from positive pools were analyzed individually by multiplex-PCR to detect the *stx-*positive colony. The characterization of the isolated strains was made by biochemical tests [23]. STEC serotyping of O and H antigens and *eae*, *aggR* and *aaiC* detection were performed as previously described [26, 27, 28].

#### Listeria monocytogenes

Twenty-five g of ground beef and one portion of sponge from each environmental sample were cultured in 225 ml and 100 ml of half Fraser broth (Becton Dickinson), respectively, for 24 h at 30°C [29]. After the pre-enrichment step, 0.1 ml was put onto 10 ml Fraser broth (Becton Dickinson) for 48 h at 37°C. Ten microliters were plated into ALOA agar (Acumedia), another 10 μl were plated into PALCAM agar (Acumedia) and incubated during 24–48 h at 37°C. The presumptive colonies were identified by Gram stain and biochemical tests [29].

#### Staphylococcus aureus

One ml from each manipulator hand sample was incubated in 9 ml Giolitti Cantoni broth (Acumedia) for 24 h at 37°C. Thereafter, 0.1 μl was streaked into Baird Parker agar (Acumedia) and incubated for 48 h at 37°C. After incubation, three presumptive colonies were selected from each plate and screened by the catalase and coagulase test, and by biochemical tests [21].

### Improvement actions and consumer information

We designed a training plan for the promotion of improvement actions in butcher shops that included collective training meetings for butchers, customized trainings for handlers, and individual counseling at the stores. Collective training meetings addressed the following topics: i) national, provincial, and local regulations about meat sale, ii) results of the evaluation stages, including risk quantification and bacteriological analysis, and iii) problems identified and possible improvements for risk mitigation. In customized trainings for handlers and individual counseling at the stores, a report with the microbiological results of samples from each butcher shop was used. Those results and the problems identified during risk assessment were used to provide recommendations about facilities, good manufacturing practices (GMP), sanitation standard operating procedures (SSOP), raw food handling and meat preservation. Also, guidelines for the implementation of improvement actions were submitted to the consideration of the butcher.

In the case of consumers, the strategy to provide information was based on a series of activities: i) training of Berisso kindergarten teachers, including information about food regulations, the results of the evaluations performed at butcher shops and recommendations to prevent foodborne diseases at home and at school; ii) delivery of information material for teachers to use in the classroom with children, kindly provided by a non-governmental organization for HUS mitigation (http://www.lusuh.org.ar/material.html); and iii) delivery of information brochures about foodborne illness prevention that the children took home.

### Verification of the impact of improvement actions

From March to December 2013, the same 86 butcher shops analyzed during the 2010–2011 evaluation period were retested to verify the program impact. Quantity and type of samples, sampling frequency and procedure, risk quantification and bacteriological analysis were performed as described previously in the evaluation stage.

### Statistical analyses

McNemar test was used to assess the impact of improvement actions on facilities, GMP, SSOP, raw food handling and meat preservation; the microbiological quality of meat sold in butcher shops, determined by the counts of mesophilic aerobic organisms, *S*. *aureus* and *E*. *coli*; and the presence or absence of *Salmonella* spp., *E*. *coli* O157:H7, non-O157 STEC and *L*. *monocytogenes* before and after implementing the improvement actions. The same statistical test was used to evaluate the impact of improvement actions on the presence of *Salmonella* spp., *E*. *coli* O157:H7, non-O157 STEC, and *L*. *monocytogenes* isolated from meat contact surfaces such as meat tables, knives, meat mincing machines and manipulator hands. Changes in risk quantification as well as in the counts of mesophilic aerobic organisms, *S*. *aureus* and *E*. *coli* in ground meat before and after the improvement actions, were evaluated using Student's paired t-test with a two-tailed distribution. In total, nine t-tests were conducted and the homogeneity of variance (Levene Test) and normality (Kolmogorov-Smirnov Test) were proved. All statistical analyses were performed using InfoStat software (Universidad Nacional de Córdoba) with a significance of 0.05.

## Results

Sixty-six shops (76.7%) were meat stores, 18 (20.9%) were butcher shops at supermarkets and two (2.3%) were corporate supermarkets selling only packaged ground beef. Ten suppliers provided meat to the 86 butcher shops.

### Comprehensive evaluation of butcher shops in the 2010–2011 period

#### Risk quantification

Risk quantification in all butcher shops resulted in 43 (50.0%) high-risk, 34 (39.5%) moderate-risk and 9 (10.5%) low-risk shops. Results for each group of five variables were as follows: situation and conditions of construction, 4.9/10.0; equipment and tools, 6.7/15.0; manipulator hands, 8.2/25.0; raw materials and products for sale, 6.9/20.0; and production flow, 14.6/30.0. Individual variables and average risk are depicted in table 1.

**Table 1:** Average risk of each group of variables during the evaluation (2010–2011) and verification (2013) periods.

Groups of variables and average risk	Individual variables^a^	Period^b^		*P*-value^c^
		2010–2011 (%)	2013 (%)	
**1.- Building situation and conditions** **Average risk (10.0)**^d^:	Access to drinking water SSOP in the water supply tank Hot water Suitable floors Suitable roofs Suitable walls Suitable windows Protected windows Adequate lighting Adequate ventilation Adequate staff changing room Adequate staff sanitation area Waste management SSOP in work environment	97.7 2.3 23.3 36.0 34.9 40.7 62.8 34.9 60.5 54.7 25.6 34.9 68.6 23.3 **4.9**	97.7 1.2 24.4 44.2 53.5 52.3 86.0 45.3 66.3 64.0 34.9 50.0 86.0 24.4 **5.8**	**<0.001**
**2.- Equipment and tools** **Average risk (15.0)**^d^:	Quantity of tools Proper conservation of tools Good conditions of tools Sufficient refrigeration equipment SSOP application on equipment and tools	77.9 75.6 65.1 66.3 34.9 **6.7**	87.2 84.9 73.3 86.0 60.5 **8.9**	**<0.001**
**3.- Handlers** **Risk average (25.0)**^d^:	Appropriate clothing Clean clothes Proper hygiene habits Health verification	45.3 48.8 17.4 29.1 **8.2**	64.0 62.8 30.2 54.7 **12.0**	**<0.001**
**4.- Raw materials and products for sale** **Average risk (20.0)**^d^:	Raw material receipt control Control of organoleptic properties in products for sale Proper conservation of raw materials and products for sale	39.5 7.0 58.1 **6.9**	67.4 4.7 82.6 **10.2**	**0.004**
**5.- Production flow** **Average risk (30.0)**^d^:	Linear flow of meat in one direction Control of cross-contamination Protection of meat products Conservation at adequate temperatures Food storage by product type Pest management Qualified personnel for handling meat	62.8 31.4 59.3 66.3 48.8 3.5 29.1 **14.6**	68.6 43.0 84.9 84.9 66. 3.5 43.0 **19.1**	**<0.001**

SSOP: Sanitation Standard Operating Procedures

^a^ Questions included in the checklist used for risk quantification (see S1 table).

^b^ Before (2010–2011) and after (2013) implementing the improvement actions.

^c^ Mc Nemar Test.

^d^ Maximum value assigned to each group of variables.

#### Bacteriological analysis

Results of microorganism enumeration in the 86 ground beef samples analyzed showed mesophilic counts <10^7^ CFU/g in 64 (74.4%) samples, *S*. *aureus* <10^3^ CFU/g in 51 (59.3%) of samples and *E*. *coli* <500 CFU/g in 52 (60.5%) samples. *Salmonella* spp. was isolated from nine (10.4%) ground beef samples, *L*. *monocytogenes* from 40 (46.5%) and *E*. *coli* O157:H7 from 10 (11.6%) samples. Non-O157 STEC was detected in 39 (45.3%) samples, though eight strains were isolated from six (7.0%) ground beef samples.

The following bacteria were isolated from the 336 environmental samples: *Salmonella* spp. (11; 3.3%), *L*. *monocytogenes* (98; 29.2%), *E*. *coli* O157:H7 (seven; 2.0%) and 24 non-O157 STEC strains (24; 7.1%). However, non-O157 STEC was detected by PCR in 47 (55.9%) meat table, 40 (47.6%) knife, 47 (55.9%) meat mincing machine, and 32 (38.1%) manipulator hands samples. Some of these STEC results have already been partially published [26]. In addition, 36 (42.9%) *S*. *aureus* strains were isolated from 84 manipulator hands samples.

### Improvement actions and consumer information

Twelve collective training meetings were organized in 61 butcher shops for 168 handlers. In addition, 86 individual training sessions for 498 handlers were held in butcher shops. In 2012, we promoted the implementation of a GMP program in all butcher shops that included the following recommendations:

1. Building situation and conditions: Improvement of ceilings, walls, floors, windows, doors, lighting and ventilation, among others. Importance of staff changing rooms, water tank SSOP and waste treatment.
2. Equipment and tools: Reinforcement of the concept of SSOP, and need for a cleaning, degreasing and disinfecting plan, particularly for refrigeration equipments. Places and conditions for storing equipment.
3. Handlers: Correct personal hygiene such as handwashing, toileting and cleaning. Staff work clothing conditioning.
4. Raw materials and products for sale: Improvement of protocols for raw material reception. Control of meat organoleptic properties.
5. Production flow: Integral GMP program. Reinforcement of concepts about correct identification of chemical and physical hazards in meat collection rooms, cross-contamination, integrated pest management, and integrated waste management. Guidelines for the implementation of improvement actions addressing butchers (available at http://www.ipcva.com.ar/files/manualcarniceros.pdf).

In addition, training about foodborne diseases prevention was provided to 76 teachers from all kindergartens in the city of Berisso. Teachers worked with 4,506 children aged 3 to 5 years using puppets, stories, games, videos and songs. All children received brochures with information on how to prevent foodborne illnesses.

### Verification of the impact of the improvement actions implemented

#### Risk quantification and evaluation

Risk was reassessed in 2013 in all butcher shops, obtaining the following results: 19 (22.1%) high-risk, 42 (48.8%) moderate-risk and 25 (29.1%) low-risk shops. All groups of variables investigated improved in 2013 compared with the 2010–1011 period (table 1). Specifically, 1) the situation and condition of buildings improved significantly, (*P*<0.001) except for the implementation of SSOP in water tanks and the work environment; 2) all the individual variables evaluated in equipment and tools also improved significantly (*P*<0.001); 3) all the aspects concerned with handlers improved as compared with that observed in the 2010–2011 period (*P*<0.001); 4) in raw materials and products for sale, with the exception of the control of organoleptic properties in products, the other variables improved considerably in 2013 compared with the 2010–1011 period (*P*<0.004); 5) most individual variables related with the production flow improved (*P*<0.001), except for integrated pest management.

#### Bacteriological analysis

Count values of the microorganisms detected in the 86 ground beef samples in both study periods are presented in table 2, as follows: mesophilic counts <10^7^ CFU/g in 72 (83.7%) samples, *S*. *aureus* <10^3^ CFU/g in 69 (80.2%) samples, and *E*. *coli* <500 CFU/g in 57 (66.3%) samples. The average count of indicator microorganism according to the microbiological criteria of the AFC was lower with respect to the 2010–2011 period. However, mesophilic (*P* = 0.102) and *E*. *coli* (*P* = 0.190) counts in ground beef were not different after the implementation of improvement actions, and *S*. *aureus* counts were lower (*P*<0.001) as compared with the beginning of the study.

**Table 2:** Microorganism counts in ground beef from butcher shops in the evaluation (2010–2011) and verification (2013) periods.

Microorganism	Period (logUFC/g)		*P*-value
	2010–2011	2013	
Mesophiles	6.49	6.32	0.102
*S*. *aureus*	1.86	0.71	<0.001
*E*. *coli*	2.10	1.81	0.190

*Salmonella* spp. was isolated from 10 (11.6%) ground beef samples, showing no statistically significant differences when compared with that observed before the implementation of improvement actions (*P* = 1.000). The proportion of samples with *L*. *monocytogenes* was lower after the implementation of improvement actions (15.1%) (*P*<0.001). The reduction in the proportion of *E*. *coli* O157:H7 isolated (three samples; 3.5%) may be considered as a tendency (*P* = 0.092). Non-O157 STEC was detected in 33 (38.4%) samples (*P* = 0.418), though 12 strains were isolated from 11 (12.8%) ground beef samples (*P* = 0.332).

From a total of 336 environmental samples analyzed, *Salmonella* spp. was isolated from five (1.5%), *L*. *monocytogenes* from 36 (10.7%), *E*. *coli* O157:H7 from two (0.6%), and 25 strains of non-O157 STEC from 23 (6.8%) samples. However, non-O157 STEC was detected in samples from 21 (25.0%) meat tables, 31 (36.9%) knives, 28 (33.3%) meat mincing machines and 25 (29.8%) manipulator hands. In addition, seven (8.3%) *S*. *aureus* strains were isolated from 84 manipulator hand samples. A significant reduction of *L*. *monocytogenes* (meat table, knife, mincing machine and hands) and *S*. *aureus* (manipulator hands) was observed after implementing the improvement actions. The presence of *E*. *coli* O157:H7 was also lower. However, the proportion of positive samples for this pathogen was low (<2.4%) in both study periods and for any environmental sample. Additionally, *E*. *coli* O157:H7 was not isolated from meat tables and knives after the implementation of improvement actions. The proportion of samples from ground beef, meat tables, knives, mincing machines and manipulator hands positive for *Salmonella* spp., *L*. *monocytogenes*, *E*. *coli* O157:H7, non-O157 STEC and *S*. *aureus* is presented in table 3. The characterization and sources of *Salmonella* and STEC strains are depicted in table 4 and S2 table, respectively.

**Table 3:** *Salmonella* spp., *L*. *monocytogenes*, *E*. *coli* O157:H7, non-O157 STEC and *S*. *aureus* isolated from ground beef and environmental samples of butcher shops in the evaluation (2010–2011) and verification (2013) periods.

Microorganism	Period ^1^	Ground beef (n = 86)		Meat tables (n = 84)		Knives (n = 84)		Mincing machines (n = 84)		Manipulator hands (n = 84)	
		Proportion % (n)	*P*-value^2^	Proportion % (n)	*P*-value^2^	Proportion % (n)	*P*-value^2^	Proportion % (n)	*P*-value^2^	Proportion % (n)	*P*-value^2^
*Salmonella* spp.	1	10.4 (9)	1.000	2.4 (2)	1.000	3.6 (3)	0.625	5.9 (5)	0.453	1.2 (1)	-
	2	11.6 (10)		2.4 (2)		1.2 (1)		2.4 (2)		0.0	
*L*. *monocytogenes*	1	46.5 (40)	<0.001	32.1 (27)	0.003	23.8 (20)	0.052	32.1 (27)	0.009	28.6 (24)	<0.001
	2	15.1 (13)		11.9 (10)		10.7 (9)		13.1 (11)		7.1 (6)	
*E*. *coli* O157:H7	1	11.6 (10)	0.092	2.4 (2)	-	1.2 (1)	-	2.4 (2)	1.000	2.4 (2)	1.000
	2	3.5 (3)		0.0		0.0		1.2 (1)		1.2 (1)	
non-O157 STEC	1	7.0 (6)	0.332	9.5 (8)	1.000	7.1 (6)	0.754	9.5 (8)	0.791	2.4 (2)	0.375
	2	12.8 (11)		10.7 (9)		4.8 (4)		7.1 (6)		4.8 (4)	
*S*. *aureus*	1	na^3^		na		na		na		42.9 (36)	<0.001
	2	na^3^		na		na		na		8.3 (7)	

^1^Period: 1 = Evaluation (2010–2011), before implementing improvement actions; 2 = Verification (2013), after implementing improvement actions;

na: not analyzed.

^2^ *P*-value = McNemar test.

^3^ See table 2.

**Table 4:** Sources of *Salmonella* spp. serovars isolated during both study periods.

Butchery	Period ^1^	Source				
#		Ground beef	Meat tables	Knives	Mincing machines	Manipulator hands
5	1		Westhampton			
7	2			Anatum	Infantis	
11	2	Newport				
		Saintpaul				
15	1	Meleagridis		Panama	Meleagridis	
17	1			Derby		
18	2	Saintpaul			Saintpaul	
20	2	Typhimurium				
24	1	Anatum			Senftenberg	
28	1	Derby				
29	1			Senftenberg		
37	2		Worthington			
39	2	Newport				
		Saintpaul				
42	2	Panama				
45	1	Derby				
51	1	Anatum			Anatum	
56	1	Newport				
58	1	Give			Give	
63	1				Newport	
	2	NT				
64	2		Montevideo			
66	1		Montevideo			
69	2	Give				
		Oranienburg				
		Montevideo				
		NT				
70	2	Typhimurium				
71	1					Newport
74	2	Anatum				
78	1	Give				
81	2	Anatum				
83	1	Derby				

^1^ Period: 1 = Evaluation (2010–2011), before implementing improvement actions; 2 = Verification (2013), after implementing improvement actions.

NT: Non-typeable.

## Discussion

The present study demonstrates the usefulness of a risk quantification method based on a simple checklist to estimate the risk of spoilage microorganisms and pathogens present in the meat sold at butcher shops. Additionally, this methodology was effective to detect improvements in both practices and facilities. The implementation of improvement actions by the butcher was determined by risk quantification, and correlated with an improvement in microbiological terms. *S*. *aureus* count and the proportion of ground beef samples positive for *L*. *monocytogenes* and *E*. *coli* O157:H7 decreased. Likewise, the proportion of environmental and manipulator hand samples positive for *L*. *monocytogenes* and *S*. *aureus*, respectively, also decreased. Regarding food manipulator hands, they have been reported as one of the most common vehicles associated with cross-contamination [15, 30]. In ground beef, we observed significant reductions in mesophilic bacteria and *E*. *coli* counts. These microorganisms can reach meat from different sources such as carcasses, manipulator hands, equipment and the environment [15, 31, 32]. Although improvement actions had an impact on some potential sources, they were not enough to generate measurable changes in these microbial groups. In addition, we could not detect reductions in the proportion of *Salmonella* spp. and non-O157 STEC-positive ground beef samples. In environmental samples, the proportion of *Salmonella* spp., *E*. *coli* O157:H7 and non-O157 STEC was reduced after the implementation of improvement actions, but changes were not statistically significant. The low proportion of positive samples for these pathogens before implementing of improvement measures could explain the lack of statistical differences, despite the observed additional reductions after their implementation.

Several studies have shown the presence of *Salmonella* spp., *E*. *coli* O157:H7, non-O157 STEC and *L*. *monocytogenes* in ground beef from butcher shops. The reported rates of contamination vary from the US, Brazil, New Zealand, Canada and China to Europe [31, 33–35]. Thus, fresh raw meat is not a sterile food, and butcher shops are not sterile places. Considering that safety is the main issue for consumers, a holistic “farm to fork” approach has been designed for the meat industry to ensure the safety of meat products. As meat may be contaminated from the slaughterhouse up to the environment of butcher shops, it is interesting to consider some aspects of the beef production chain.

Fecal contamination of bovine carcasses can occur during the slaughter process. Rhoades et al. [31] reviewed the proportion of samples testing positive for *Salmonella* spp. (12.7–25.1%), *E*. *coli* O157:H7 (10.1–43.4%) and *L*. *monocytogenes* (1.1–4.8%) in raw beef from different countries at pre-evisceration. In Argentina, Masana et al. [36] reported the presence of *E*. *coli* O157:H7 (2.6%) and non-O157 STEC (9.0%) in 822 carcasses from eight slaughterhouses. In addition, Etcheverria et al. [37] reported 12.3–18.6% of non-O157 STEC in carcasses. Rhoades et al. [31] summarized the presence of *Salmonella* spp. (3.5–7.5%), *E*. *coli* O157:H7 (0.0–0.5%), non-O157 STEC (5.7–16.8%) and *L*. *monocytogenes* (1.6–19.6%) in ground beef from the US. Similar data were reported in Europe for *Salmonella* spp. (0.4–3.5%), *E*. *coli* O157:H7 (0.0–2.8%), non-O157 STEC (1.1–15.5%) and *L*. *monocytogenes* (1.2–11.8%) [31]. These studies included meat products, but none of them analyzed environmental samples.

In the present work, the proportion of ground beef samples testing positive for *Salmonella* spp. was 10.4% in the 2010–2011 period and 10.6% in 2013, lower than the 17.0% reported in China [34], and higher than the 0.42% reported in the US [35]. Food equipment has been recognized as an important vehicle of contamination throughout the meat supply chain [38, 39]. In this regard, we isolated different *Salmonella* serovars (Meleagridis, Anatum, Give and Saintpaul) from ground beef and mincing machines of three butcher shops, during the 2010–2011 period and one in 2013. Probably, the poor sanitation of mincing machines was the origin of contamination. Several *Salmonella* serovars, such as Anatum, Give, Montevideo, Newport, and Senftenberg have been identified in different foods and reported to form biofilms [40]. During 2013, the low proportion of *Salmonella* in environmental samples proved that cross-contamination was reduced. On the other hand, *S*. Typhimurium was isolated only from ground beef of two different butcher shops after implementing the improvement actions. Contamination may have had the same origin because both butcher shops purchased meat from the same abattoir. Most *Salmonella* serovars isolated in this work, such as Anatum, Derby, Give, Infantis, Montevideo, Newport, Oranienburg, Panama, Saintpaul, Senftenberg and Typhimurium, are associated with human diseases worldwide [41–44]. However, the local sanitary authorities did not report any disease outbreak or case caused by these serovars in Berisso during the study period.

The proportion of ground beef samples testing positive for *E*. *coli* O157:H7 (11.6% and 3.5% in 2010–2011 and 2013, respectively) was similar to the 15.0% reported by Llorente et al. [45] for ground beef from butcher shops in Argentina. On the other hand, the range of *E*. *coli* O157:H7 isolation in ground beef from the US, Brazil, Italy and the United Kingdom was 0.3–0.8% [31, 46, 47]. The proportion of environmental samples testing positive for *E*. *coli* O157:H7 was 2.0% in the 2010–2011 period and 0.6% in 2013. Before implementation of the improvement actions, *E*. *coli* O157:H7 with the same genotypic profile was isolated from ground beef, meat table and mincing machine, ground beef and mincing machine, and meat table, mincing machine and manipulator hands, respectively, in three butcher shops. Just like *Salmonella* spp., *E*. *coli* O157:H7 has the ability to form biofilm [15, 32, 39], and our findings reconfirmed the absence of SSOP and GMP in the 2010–2011 period. During 2013, cross-contamination with *E*. *coli* O157:H7 between the environment and ground beef was not detected.

The proportion of ground beef samples testing positive for *stx* gene was 45.3% in 2010–2011 and 38.4% in 2013. Whereas worldwide such proportion is around 2.4–30% [48–51], in Argentina, it has been reported in the range of 18.4–40.7% [37, 45, 52], similar to that found in our study. Regarding environmental samples, *stx* gene proportion was 49.4% during the 2010–2011 period and 31.2% in 2013, demonstrating a clear reduction after implementing improvement actions. However, the percentage of non-O157 STEC isolated from ground beef and environmental samples was 7.0–12.8% and 6.8–7.1% in each period. In Spain, Australia, Canada, the Netherlands and Switzerland, non-O157 STEC percent isolation from ground beef samples was 1.0–16.0% [31, 53, 54], as compared with the 11.0–18.2% reported for Argentina [45]. Such difference could be due to the fact that there is no single method or combination of isolation methods capable of identifying all STEC serogroups [55].

In Argentina, HUS is endemic. Annually, between 300 and 500 new HUS cases are reported [56], with an average annual incidence rate of 1.1 case/100,000 inhabitants and 8.4 cases/100,000 children under 5 years of age in the period 2010–2015 [57].

The frequency of STEC isolation from acute diarrhea, bloody diarrhea or HUS cases was as follows: *E*. *coli* O157:H7, 74.9%; O145[H27; H-; NT], 13.3%; O121[H19], 2.2%; O26 [H2;11; NT], 1.8%; O174[H8; 21; 28; H-], 1.1%; O111[H-, NT], 0.6%; O8:H19, 0.5%; among other serotypes. Most of these isolates (94.3%) harbored *stx* and *eae* genes [4]. According to the EFSA [58], there is not a unique combination of markers that define pathogenic STEC strains. However, *stx*_2_/*eae* and *stx*_2_/*aaiC*/*aggR* strains were associated with a higher potential risk of causing severe illness than other combinations of virulence genes [58]. In this study, all non-O157 STEC strains were *aaiC*/*aggR*-negative and 96.5% were *eae*-negative. Among these strains, O26:H11 (*stx*_1_/*eae*) and O109:H25 (*stx*_2_/*eae*) were isolated from ground beef and mincing machines during the verification stage. In the 2010–2011 period, 43.9% of these strains isolated from ground beef and environmental samples corresponded to the main non-O157 STEC serotypes associated with illness cases in Argentina. After the implementation of improvement actions, the presence of these serotypes was reduced to 13.5%. Even though their detection at butcher shops poses a potential public health risk, no HUS cases were reported in Berisso during the 2010–2013 period, as informed the local sanitary authorities.

Although meat can get contaminated with *L*. *monocytogenes* at the slaughterhouse [59, 60] the main source of contamination could be at butcher shops since *L*. *monocytogenes* can colonize the environment by biofilm formation [12]. Papadopoulou et al. [32] demonstrated that *L*. *monocytogenes* were transferred to ground beef during meat mincing. In our study, in the 2010–2011 period, the proportion of ground beef and environmental samples testing positive for *L*. *monocytogenes* was 46.5% and 29.2%, respectively. In 2013, such proportion was reduced to 15.1% and 10.7%, respectively. We assume that this reduction was due to the implementation of actions aimed at improving sanitation. However, it was not possible to completely eliminate *L*. *monocytogenes* from the environment of butcher shops, probably because biofilms are difficult to remove and additional physical and chemical mechanisms would be required to reduce their occurrence [12].

The high prevalence of pathogenic bacteria in butcher shops of Berisso was significantly reduced or improved after implementing SSOP in all retails. The present results demonstrate that the low initial quality of ground beef could be modified through the application of improvement actions. Since, there are no previous reports about the microbiological status of ground beef and the environment of butcher shops in Argentina, we could not compare our results with those of other country regions. However, we consider that systematic monitoring and improvement actions, work training and food handler awareness, improved both product and environment quality, regardless of the individual situation of each region.

Retail meat is often associated with direct exposure to pathogens and bacteria, and with cross-contamination of the kitchen environment and ready-to-eat foods. However, many foodborne illnesses have their origin in the household kitchen, and it is at this level that the most effective controls can often be applied. This emphasizes the importance of consumer education and communication of information on emerging foodborne hazards to consumers. Considering that Argentina has reported a high incidence of HUS in children under 5 years, and whereas in the period 2002–2009 nine of the 12 foodborne outbreaks caused by STEC were recorded in kindergartens [4], educating children on safe food handling is critical to prevent foodborne diseases today and in the future. Other interventions are needed to reduce the presence of pathogens on beef products [61], and consumers should continue to be vigilant about the preparation of ground beef products and the prevention of cross-contamination at home. Additionally, consumption of undercooked meat products and cross-contamination during food handling and preparation must be avoided to ensure food safety at home and in the food service.

## Conclusions

This study focused on the effective implementation of strategies to reduce the presence of foodborne bacterial pathogens at butcher shops. The risk quantification technique was useful to identify relevant facts that should be corrected in order to improve the microbiological quality of meat. This routine methodology would help identify the areas or practices that should be improved, without the need of taking many meat and environmental samples for microbiological analysis. Although we could reduce ground beef contamination with *Salmonella* spp., *E*. *coli* O157:H7, non-O157 STEC and *L*. *monocytogenes*, identification of ground beef negative for these bacteria is circumstantial if the environment where meat is processed is not controlled. We could also reduce environmental contamination after training handlers on the basis of the problems identified in their own butcher shops. This study confirms the feasibility of implementing a comprehensive risk management program in butcher shops, and the importance of disseminating the results through information campaigns targeting the community. However, it is necessary to expand collaborative efforts to improve the safety of foodstuff at the retail level and at home to prevent foodborne diseases.

## Supporting Information

S1 Table Cheklist used for risk quantification. (DOC)

S2 Table Sources and characterization of STEC isolated during both study periods. (DOC)
