# Supplementary material for: Comprehensive Evaluation and Implementation of Improvement Actions in Butcher Shops
Source: PLoS One. 2016 Sep 12;11(9):e0162635. doi: 10.1371/journal.pone.0162635 (PMC5019392; doi:10.1371/journal.pone.0162635)
Supplement: S1 Table — (DOC) [file pone.0162635.s001.doc]

| **Variable number** | **Description** | **Qualification** | | |
| --- | --- | --- | --- | --- |
| **1** | **Situation and condition of building** | **YES** | **NO** | **NA** |
| 1.1 | Waste in the exterior area | 4 | 0 | 4 |
| 1.2 | 1.2.1. Suitable floors | 1 | 0 | 1 |
|  | 1.2.2. Clean floors | 1 | 0 | 1 |
| 1.3 | 1.3.1. Suitable roofs | 1 | 0 | 1 |
|  | 1.3.2. Clean roofs | 1 | 0 | 1 |
| 1.4 | 1.4.1. Suitable walls | 1 | 0 | 1 |
|  | 1.4.2. Clean walls | 1 | 0 | 1 |
| 1.5 | Suitable windows | 1 | 0 | 1 |
| 1.6 | Protected windows | 4 | 0 | 4 |
| 1.7 | Adequate lighting | 1 | 0 | 1 |
| 1.8 | Adequate ventilation | 1 | 0 | 1 |
| 1.9 | Adequate staff sanitation area:  1.9.1.- According to the Argentinian Food Code | 2 | 0 | 2 |
|  | 1.9.2.- Complete and clean | 4 | 0 | 4 |
| 1.10 | Adequate staff changing room:  1.10.1.- According to the Argentinian Food Code | 1 | 0 | 1 |
|  | 1.10.2.- Complete and clean | 2 | 0 | 2 |
| 1.11 | Lavatory in the food handling area:  1.11.1.- Quantity, location, and adequate installation | 2 | 0 | 2 |
|  | 1.11.2.- Complete and clean. | 4 | 0 | 4 |
| 1.12 | Access to drinking water | 8 | 0 | 8 |
| 1.13 | Water supply tank:  1.13.1.- Adequate water pressure | 4 | 0 | 4 |
|  | 1.13.2.- SSOP in the water supply tank | 8 | 0 | 8 |
| 1.14 | 1.14.1.- Waste management | 4 | 0 | 4 |
|  | 1.14.2.- SSOP in the work environment | 2 | 0 | 2 |
| 1.15 | Hot water | 2 | 0 | 2 |
|  | C1= Qualification Group 1  TS1 x 10  C1= --------------  60 – TNA1  TS1= Total YES obtained  TNA= Total NA obtained  K1= 60 (Constant Group 1)  I 1= 10 (Importance value for Group 1) |  |  |  |

| **Variable number** | **Description** | **Qualification** | | |
| --- | --- | --- | --- | --- |
| **2** | **Equipment and tools** | **YES** | **NO** | **NA** |
| 2.1 | 2.1.1.- Quantity of equipment | 2 | 0 | 2 |
|  | 2.1.2.- Proper equipment | 4 | 0 | 4 |
| 2.2 | Tools |  |  |  |
|  | 2.2.1.- Quantity of tools | 2 | 0 | 2 |
|  | 2.2.2.- Proper conservation of tools | 4 | 0 | 4 |
| 2.3 | Furniture |  |  |  |
|  | 2.3.1.- Proper conservation of furniture | 2 | 0 | 2 |
|  | 2.3.2.- Clean furniture | 4 | 0 | 4 |
| 2.4 | Refrigeration equipment:  2.4.1.- Sufficient refrigeration equipment | 8 | 0 | 8 |
|  | 2.4.2.- Clean refrigeration equipment | 8 | 0 | 8 |
| 2.5 | SSOP application on equipment and tools | 8 | 0 | 8 |
| 2.6 | Adequate location of equipments and tools | 8 | 0 | 8 |
|  | C2= Qualification Group 2  TS2= Total YES obtained  TNA= Total NA obtained  K2= 50 (Constant Group 2)  I 2= 15 (Importance value for Group 2) | TS2 x 15  C2= --------------  50 – TNA2 |  |  |

| **Variable number** | **Description** | **Qualification** | | |
| --- | --- | --- | --- | --- |
| **3** | **Handlers** | **YES** | **NO** | **NA** |
| 3.1 | Clothing  3.1.1.- Appropriate clothing. | 2 | 0 | 2 |
|  | 3.1.2.- Clean clothes | 8 | 0 | 8 |
| 3.2 | Proper hygiene habits | 8 | 0 | 8 |
| 3.3 | Good manufacture practices according to the Argentinian Food Code | 4 | 0 | 4 |
| 3.4 | Health verification | 10 | 0 | 10 |
|  | C3= Qualification Group 3  TS3= Total YES obtained  TNA= Total NA obtained  K3= 32 (Constant Group 3)  I 1= 25 (Importance value for Group 3) | TS3 x 25  C3= --------------  32 – TNA3 |  |  |

| **Variable number** | **Description** | **Qualification** | | |
| --- | --- | --- | --- | --- |
| **4** | **Raw materials and products for sale** | **YES** | **NO** | **NA** |
| 4.1 | Raw material receipt control | 4 | 0 | 4 |
| 4.2 | Control of organoleptic properties in products for sale | 8 | 0 | 8 |
| 4.3 | Proper conservation of raw materials and products for sale | 6 | 0 | 6 |
| 4.4 | Sealed parts are preserved | 6 | 0 | 6 |
|  | C4= Qualification Group 4  TS4= Total YES obtained  TNA= Total NA obtained  K4= 24 (Constant Group 4)  I 4= 20 (Importance value for Group 4) | TS4 x 20  C4= --------------  24 – TNA4 |  |  |

| **Variable number** | **Description** | **Qualification** | | |
| --- | --- | --- | --- | --- |
| **5** | **Production flow** | **YES** | **NO** | **NA** |
| 5.1 | Linear flow of meat in one direction | 4 | 0 | 4 |
| 5.2 | Control of cross-contamination | 8 | 0 | 8 |
| 5.3 | Protection of meat products | 4 | 0 | 4 |
| 5.4 | Identification and adequate conservation of toxic and chemical products | 4 | 0 | 4 |
| 5.5 | Meat conservation:  5.5.1.- Conservation at adequate temperatures | 8 | 0 | 8 |
|  | 5.5.2.- Food storage by product type | 4 | 0 | 4 |
| 5.6 | Waste is immediately removed | 4 | 0 | 4 |
| 5.7 | Control of organoleptic properties in all final products | 4 | 0 | 4 |
| 5.8 | Adequate exhibition of products for sale | 2 | 0 | 2 |
| 5.9 | Pest management | 4 | 0 | 4 |
| 5.10 | Qualified personnel for handling meat | 4 | 0 | 4 |
| 5.11 | Meat is ground at the time of selling according the Argentinian Food Code | 3 | 0 | 3 |
|  | CB5= Qualification Group 5  TS5= Total of qualifications YES obtained  TNA= Total of qualifications NA obtained  K5= 53 (Constant Group 5)  I 5= 30 (Importance value for Group 5) | TS5 x 30  C5= --------------  53 – TNA5 |  |  |

Evaluation: For each variable analyzed, mark with a circle YES, NO or Not Applicable (NA) as appropriate.

**Qualification:** Once the audit is completed, values from each group of variables are added up. Note: high-risk, 0-40; moderate-risk, 41-70; low-risk, 71-100.
