# Supplementary material for: Comprehensive Evaluation and Implementation of Improvement Actions in Butcher Shops
Source: PLoS One. 2016 Sep 12;11(9):e0162635. doi: 10.1371/journal.pone.0162635 (PMC5019392; doi:10.1371/journal.pone.0162635)
Supplement: S2 Table — (DOC) [file pone.0162635.s002.doc]

| **Butcher shop** | **Period**^a^ | **Sources** | | | | | | | | | |
| --- | --- | --- | --- | --- | --- | --- | --- | --- | --- | --- | --- |
| **#** |  | **Ground** **beef** | | **Meat tables** | | **Knives** | | **Mincing machines** | | **Manipulator hands** | |
|  |  | Serotype | Genotype | Serotype | Genotype | Serotype | Genotype | Serotype | Genotype | Serotype | Genotype |
| 1 | 1 |  |  |  |  |  |  | O174:H28^b^ | *stx*_1_/*stx*_2_ |  |  |
| 2 | 1 |  |  |  |  |  |  | O178:H19 | *stx*_2_ |  |  |
| 4 | 1 | O157:H7 | *stx*_1_/*stx*_2_/*eae* | O157:H7 | *stx*_1_/*stx*_2_/*eae* |  |  | O157:H7 | *stx*_1_/*stx*_2_/*eae* | O157:H7 | *stx*_2_/*eae* |
| 5 |  |  |  |  |  |  |  |  |  | O8:H19^b^ | *stx*_2_ |
| 10 | 1 |  |  | O174:H28^b^ | *stx*_2_ |  |  |  |  |  |  |
| 11 | 1 |  |  | O44:Hnt | *stx*_2_ |  |  |  |  |  |  |
| 17 | 1 | O157:H7 | *stx*_2_/*eae* |  |  |  |  | O157:H7 | *stx*_2_/*eae* |  |  |
|  | 2 | O163:NM | *stx*_2_ |  |  |  |  |  |  |  |  |
| 20 | 1 | O157:H7 | *stx*_2_/*eae* |  |  |  |  |  |  |  |  |
|  |  | O8:H19^b^ | *stx*_2_ |  |  |  |  |  |  |  |  |
|  |  | Ont:H19 | *stx*_1_/*stx*_2_ |  |  |  |  |  |  |  |  |
|  |  | O8:H19^b^ | *stx*_1_/*stx*_2_ |  |  |  |  |  |  |  |  |
| 23 | 1 |  |  | O8:H19^b^ | *stx*_1_/*stx*_2_ |  |  |  |  |  |  |
|  | 2 |  |  | Ont:H7 | *stx*_2_ |  |  |  |  |  |  |
| 24 | 2 |  |  | O130:H11 | *stx*_1_/*stx*_2_ | Ont:H28 | *stx*_2_ |  |  |  |  |
| 27 | 1 |  |  | O157:H7 | *stx*_2_/*eae* | O157:H7 | *stx*_2_/*eae* |  |  | O157:H7 | *stx*_2_/*eae* |
|  |  |  |  | O79:H19 | *stx*_2_ |  |  |  |  |  |  |
| 28 | 2 | O26:H11^b^ | *stx*_1_/*eae* |  |  |  |  |  |  |  |  |
| 29 | 1 |  |  |  |  | O8:H19^b^ | *stx*_1_/*stx*_2_ | Ont:H18 | *stx*_1_/*stx*_2_ |  |  |
| 31 | 1 | O157:H7 | *stx*_2_/*eae* |  |  |  |  |  |  |  |  |
| 32 | 1 | O157:H7 | *stx*_2_/*eae* |  |  |  |  | O178:H19 | *stx*_2_ |  |  |
| 33 | 2 |  |  | O116:H21 | *stx*_2_ |  |  |  |  |  |  |
| 36 | 2 | Ont:H28 | *stx*_2_ |  |  |  |  |  |  |  |  |
| 37 | 2 |  |  |  |  |  |  |  |  | Ont:H21 | *stx*_2_ |
| 40 | 1 |  |  |  |  |  |  | O174:H21^b^ | *stx*_2_ |  |  |
| 41 | 1 | O41:H14 | *stx*_2_ |  |  |  |  |  |  |  |  |
| 44 | 1 | O157:H7 | *stx*_2_/*eae* |  |  |  |  |  |  |  |  |
|  | 2 | O174:H2^b^ | *stx*_2_ | Ont:H19 | *stx*_2_ |  |  | O157:H7 | *stx*_2_/*eae* |  |  |
|  |  |  |  | Ont:H49 | *stx*_2_ |  |  | Ont:H19 | *stx*_2_ |  |  |
| 45 | 1 | O157:H7 | *stx*_2_/*eae* |  |  |  |  |  |  |  |  |
| 46 | 1 | O174:H21^b^ | *stx*_2_ |  |  |  |  |  |  |  |  |
| 47 | 2 | O157:H7 | *stx*_2_/*eae* | O174:H28^b^ | *stx*_1_/*stx*_2_ | Ont:H19 | *stx*_1_ |  |  |  |  |
| 49 | 2 | O141:H49 | *stx*_2_ |  |  |  |  | O109:H25 | *stx*_2_/*eae* |  |  |
|  |  | Ont:H49 | *stx*_2_ |  |  |  |  |  |  |  |  |
| 51 | 1 |  |  |  |  | O116:H21 | *stx*_2_ |  |  |  |  |
| 52 | 1 | O157:H7 | *stx*_2_/*eae* |  |  |  |  |  |  |  |  |
| 53 | 2 | O64:H20 | *stx*_2_ |  |  |  |  |  |  |  |  |
| 56 | 2 |  |  |  |  |  |  |  |  | O157:H7 | *stx*_2_/*eae* |
| 57 | 1 |  |  | O8:H19^b^ | *stx*_1_/*stx*_2_ |  |  |  |  |  |  |
|  | 2 | Ont:H19 | *stx*_2_ |  |  |  |  |  |  | O116:H49 | *stx*_2_ |
|  |  | O157:H7 | *stx*_2_/*eae* |  |  |  |  |  |  |  |  |
| 58 | 2 |  |  |  |  |  |  |  |  | O21:H21 | *stx*_1_ |
| 59 | 1 |  |  |  |  | Ont:H21 | *stx*_2_ | Ont:H7 | *stx*_2_ |  |  |
| 61 | 2 | O178:H19 | *stx*_1_/*stx*_2_ | O178:H19 | *stx*_1_/*stx*_2_ | O178:H19 | *stx*_1_/*stx*_2_ | O171:H14 | *stx*_2_ |  |  |
|  |  |  |  | O171:H14 | *stx*_2_ |  |  |  |  |  |  |
| 62 | 1 |  |  |  |  | O130:H21 | *stx*_2_ |  |  |  |  |
| 63 | 1 |  |  |  |  |  |  | O79:H19 | *stx*_2_ |  |  |
|  | 2 |  |  | O141:H49 | *stx*_1_/*stx*_2_ |  |  |  |  |  |  |
| 64 | 2 | O157:H7 | *stx*_2_/*eae* |  |  |  |  |  |  |  |  |
| 69 | 1 | O157:H7 | *stx*_2_/*eae* |  |  |  |  |  |  |  |  |
| 70 | 2 | O91:H21 | *stx*_2_ |  |  |  |  | O91:H21 | *stx*_2_ |  |  |
| 73 | 1 |  |  |  |  |  |  |  |  | O174:NM^b^ | *stx*_2_ |
| 74 | 2 |  |  |  |  |  |  | Ont:H19 | *stx*_2_ | O174:H21^b^ | *stx*_2_ |
| 75 | 2 |  |  |  |  |  |  |  |  | Ont:NM | *stx*_2_ |
| 76 | 1 |  |  | O174:H28^b^ | *stx*_2_ | O174:H28^b^ | *stx*_2_ |  |  |  |  |
| 77 | 2 |  |  | Ont:H7 | *stx*_2_ |  |  |  |  |  |  |
| 79 | 1 |  |  |  |  | Ont:Hnt | *stx*_2_ |  |  |  |  |
| 81 | 1 | Ont:H7 | *stx*_2_ | Ont:H7 | *stx*_2_ |  |  |  |  |  |  |
|  | 2 |  |  | Ont:H19 | *stx*_2_ |  |  |  |  |  |  |
| 82 | 1 |  |  | Ont:H7 | *stx*_2_ |  |  |  |  |  |  |
|  | 2 | O113:H21 | *stx*_2_ |  |  |  |  |  |  |  |  |
| 83 | 1 | O8:H19^b^ | *stx*_2_ |  |  |  |  |  |  |  |  |
| 84 | 2 | Ont:Hnt | *stx*_2_ |  |  |  |  |  |  |  |  |
| 85 | 1 | O157:H7 | *stx*_2_/*eae* |  |  |  |  | O113:H21 | *stx*_1_/*stx*_2_ |  |  |
|  |  | O113:H21 | *stx*_1_/*stx*_2_ |  |  |  |  |  |  |  |  |
|  | 2 |  |  |  |  | O174:H21^b^ | *stx*_2_ |  |  |  |  |

^a^ 1, Evaluation (2010-2011), before implementing improvement actions; 2, Verification (2013), after implementing improvement actions.

^b^ Main non-O157 STEC serotypes associated with illness cases in Argentina.

nt: Nontypeable. NM: Nonmotile. Genotype: *stx*_1_, *stx*_2_, *eae*, *aaiC* and *aggR* genes were screened. All STEC strains were negative for *aggR* and *aaiC* genes.
